# Effect of CpG Depletion of Vector Genome on CD8^+^ T Cell Responses in AAV Gene Therapy

**DOI:** 10.3389/fimmu.2021.672449

**Published:** 2021-05-31

**Authors:** Thais B. Bertolini, Jamie L. Shirley, Irene Zolotukhin, Xin Li, Tsuneyasu Kaisho, Weidong Xiao, Sandeep R. P. Kumar, Roland W. Herzog

**Affiliations:** ^1^ Herman B Wells Center for Pediatric Research, Indiana University School of Medicine, Indianapolis, IN, United States; ^2^ Department Pediatrics, University of Florida, Gainesville, FL, United States; ^3^ Department of Immunology, Institute of Advanced Medicine, Wakayama Medical University, Wakayama, Japan; ^4^ Laboratory for Inflammatory Regulation, RIKEN Center for Integrative Medical Sciences, Yokohama, Japan

**Keywords:** CD8^+^ T cell, dendritic cells, CpG, adeno-associated virus, TLR9, gene therapy, hemophilia

## Abstract

Adeno associated viral (AAV) vectors have emerged as a preferred platform for *in vivo* gene replacement therapy and represent one of the most promising strategies to treat monogenetic disorders such as hemophilia. However, immune responses to gene transfer have hampered human gene therapy in clinical trials. Over the past decade, it has become clear that innate immune recognition provides signals for the induction of antigen-specific responses against vector or transgene product. In particular, TLR9 recognition of the vector’s DNA genome in plasmacytoid dendritic cells (pDCs) has been identified as a key factor. Data from clinical trials and pre-clinical studies implement CpG motifs in the vector genome as drivers of immune responses, especially of CD8^+^ T cell activation. Here, we demonstrate that cross-priming of AAV capsid-specific CD8^+^ T cells depends on XCR1^+^ dendritic cells (which are likely the main cross-presenting cell that cooperates with pDCs to activate CD8^+^ T cells) and can be minimized by the elimination of CpG motifs in the vector genome. Further, a CpG-depleted vector expressing human coagulation factor IX showed markedly reduced (albeit not entirely eliminated) CD8^+^ T cell infiltration upon intramuscular gene transfer in hemophilia B mice when compared to conventional CpG^+^ vector (comprised of native sequences), resulting in better preservation of transduced muscle fibers. Therefore, this deimmunization strategy is helpful in reducing the potential for CD8^+^ T cell responses to capsid or transgene product. However, CpG depletion had minimal effects on antibody responses against capsid or transgene product, which appear to be largely independent of CpG motifs.

## Introduction

Adeno associated virus (AAV) has emerged as the preferred platform for *in vivo* gene replacement therapy and represents one of the most promising strategies to treat monogenetic disorders such as hemophilia. Two AAV-based gene therapies have received regulatory approval and many more are currently under investigation in late-stage clinical trials (1, 2). However, adaptive immune responses directed against the AAV capsid and the transgene product continue to limit long-term clinical success (3–6). These include cytotoxic T lymphocytes (CTLs) that eliminate transduced cells by recognizing peptides derived from the AAV capsid and activated B cells that produce antibodies capable of neutralizing the therapeutic transgene product. In clinical trials, immunosuppressive drugs are frequently administered to mitigate immune mediated rejection of AAV and preserve therapeutic gene expression. However, these drugs come with significant safety concerns and inadequately address AAV-associated immune responses and immunotoxicities. Thus, there is a growing interest in developing AAV vectors that are devoid of immunogenic features and can escape immune detection all together.

Innate immune sensing is critical for the initial detection of microbes and conditions downstream adaptive immune responses. Antigen presenting cells like macrophages and dendritic cells (DCs), express a diverse repertoire of pattern recognition receptors such as toll-like receptors (TLRs) that each recognize distinct, evolutionarily conserved, pathogen associated molecular patterns (PAMPs). Signaling through these receptors triggers a cascade of immunoregulatory events that balance the decision to respond with tolerance or immunity. Initial innate immune detection of AAV vectors occurs *via* toll-like receptor 9 (TLR9) (7, 8). TLR9 is an endosomal DNA sensor that recognizes unmethylated cytosine-phosphate-guanine (CpG) motifs commonly present in viral and bacterial DNA genomes. Importantly, TLR9 sensing of AAV vector genomes has been shown to be required for anti-capsid CD8^+^ T cell responses (9–14). Specifically, TLR9 signaling in plasmacytoid dendritic cells (pDCs) is required for adaptive immune responses to AAV. Type I interferons (T1 IFN) produced downstream of TLR9 in pDCs are necessary to activate conventional DCs (cDCs) which process and present AAV capsid derived antigens on MHC class I (MHC I) to prime anti-capsid CD8^+^ T cells. Indeed, both TLR9 and T1 IFNs (and also CD4^+^ T help) are requisite for anti-capsid CD8^+^ T cell priming (9, 15). Thus, CpG motifs present in the DNA genome of AAV gene therapy vectors are key determinants of vector immunogenicity.

Evidence from early clinical trials, along with subsequent laboratory studies, suggest that CD8^+^ T cell responses against AAV capsid target transduced hepatocytes (16–18). Moreover, a recent meta-analysis of clinical trials using hepatic AAV gene transfer concluded that vectors rich in CpG motifs results in failure to achieve sustained expression, owing to activation of capsid specific CD8^+^ T cells (19–22). Therefore, elimination of CpG motifs has become a feature of more recent vector designs. Additionally, Faust et al. demonstrated that use of a CpG depleted expression cassette ablated CTL responses to the β-galactosidase transgene upon AAV gene transfer in mice (23), suggesting this strategy may reduce the risk of immune response directed against the transgene product.

Here, we demonstrate that depletion of CpG motifs from the AAV expression cassette substantially reduces cross-priming of capsid specific CD8^+^ T cells in mice. We further show in hemophilia B mice that CD8^+^ T cell responses against the therapeutic transgene product, coagulation factor IX (FIX), are dramatically reduced using the CpG-depleted vector construct compared to native sequences. Interestingly, this approach was not useful to prevent antibody formation against capsid or transgene product.

## Materials And Methods

### Mice

Wild type (WT) C57BL/6 mice were purchased from Jackson Laboratories (Bar Harbor, ME). XCR1^+/DTRvenus^ mice were obtained from RIKEN Center for Integrative Medical Sciences. In XCR1^+/DTRvenus^ mice, coding region of the *Xcr1* gene was replaced with a genetic cassette encoding diphtheria toxin receptor (DTR) and reporter protein “Venus” (a yellow fluorescent protein) (24, 25). Expression of DTR under XCR1 promoter allowed specific depletion of XCR1^+^ DCs by administration of diphtheria toxin (DT) whereas expression of fluorescent protein allowed their easy detection and tracking. Hemophilia B (C3H/HeJ-F9-/Y) mice with a targeted deletion of murine *F9* gene had been bred on the C3H/HeJ background as published (24–26). All animals were maintained at laboratory animal resource center facility at Indiana University–Purdue University, Indianapolis (IUPUI). All animal experiments were performed as per the guidelines of Institutional Animal Care and Use Committee (IACUC). Male mice, 6 to 8 weeks of age were used. The specific number of mice in each cohort is indicated in each figure with a minimum of four mice per group.

### AAV Vector

AAV1 and AAV2 serotype were used to perform these studies. To assess capsid specific CD8^+^ T cells a surrogate epitope SIINFEKL (a dominant CD8^+^ T cell epitope derived from ovalbumin) was cloned into the AAV2 capsid HI loop (9). Two gene cassettes [one with native CpG sequence (27) and other with CpG-depleted sequence] of human coagulation factor IX (hFIX) containing Padua mutation were used in these studies. CpG depleted sequence of hFIX with Padua mutation was custom synthesized by Invivogen (San Diego, CA, USA) and is provided in **Supplementary Figure 1**. Both cassettes were under the transcriptional control of a cytomegalovirus (CMV) immediate early gene 1 enhancer/human elongation factor-1α (EF1α) promoter combination and had SV40 polyadenylation sequence (Invivogen). The naturally occurring Padua mutation represents a single amino acid change (R338L) in human FIX that results in a ~1 log increased specific activity, resulting for higher efficacy in gene therapy per FIX antigen level (28). All AAV vectors used in this study were single stranded (ss) and were produced by triple transfection of HEK-293 cells. All AAV vectors were purified by double iodixanol gradient centrifugation and titers determined by quantitative PCR (29).

### Animal Procedures

XCR1^+^ DCs were depleted by administering 1 µg of DT (Millipore, Massachusetts, USA) to XCR1^+/DTRvenus^ mice. CD103^+^ DCs were neutralized by administration of 150 µg of anti-CD103 antibody (BioXCell, Lebanon, NH) to C57BL6 wild type (WT) mice. In order to maintain the depletion of XCR1^+^ DCs and neutralization of CD103^+^ DCs, DT was administered on day −1, 3, and 7, and anti-CD103 antibody was administered on day −1, 3, 7, and 10, both *via* intraperitoneal (i.p) route. For all experiments, mice were injected intramuscularly (i.m) into the quadricep muscle with 50 µL of AAV containing 1 × 10^11^ vg. Post AAV administration mice were bled at different time points *via* retro-orbital plexus using heparinized capillaries. Blood from hemophilia B mice was collected using untreated capillaries in to 0.38% sodium citrate buffer. Hemophilia B mice were euthanized at the end of experiment (28 days post AAV administration) and quadricep anterior muscle was harvested. Excised muscles were cryo-protected in optimal cutting temperature media using liquid N_2_-cooled 2-methylbutane (30).

### Flow Cytometry

To quantify capsid specific CD8^+^ T cells, flow cytometry was performed on peripheral blood mononuclear cells (PBMC) using antibodies to CD3 (17A2), CD8a (53–6.7) and MHC I tetramer (H-2Kb-SIINFEKL, MBL International, Woburn, MA, USA) (15). To assess depletion of XCR1^+^ DCs, spleens were pretreated with collagenase D (Roche, Basel, Switzerland) at 2 mg/mL for 20 min at 37°C. Single cell suspension of splenocytes were prepared and stained with CD11c (N418), XCR1 (ZET), and CD8a (53–6.7) antibodies (BioLegend, San Diego, CA). Flow cytometry data was acquired on the Fortessa flow cytometer (BD Biosciences) and analyzed using FCS express 7 (DeNovo Software, Los Angeles, CA).

### Immunohistochemistry

Immunohistochemistry was performed on cryo-sections of quadricep muscle, as previously described (30). Briefly, cryosections (10 μm) of muscle were fixed in pre-cooled acetone at −20°C, blocked with 5% donkey serum (Sigma, St. Louis, MO) and stained with rat anti-CD8α (eBioscience) and goat anti-hFIX (Affinity Biologicals, Ontario, CA) antibodies at room temperature. Secondary antibodies, donkey anti-rat conjugated to Alexa Fluor 488 and donkey anti-goat conjugated to Alexa Fluor 568 (Life Technologies, Carlsbad, CA, USA) were used for detection. Sections were mounted using Prolong Diamond antifade with DAPI mounting media (Invitrogen, Carlsbad, CA). Mounted sections were stored protected from light at 4°C until visualization and image acquisition.

### Image Acquisition and Analyses

Slides were scanned and digitized using an Axio observer 7 Zeiss microscope (Carl Zeiss Microscopy, LLC, Thornwood, NY). Whole muscle sections were captured with a 40× objective, using the tiles option. Infiltrating CD8^+^ T cell were quantified on whole muscle sections (area average 612.2519) using Fiji-ImageJ software after generating a grid (area per point, 1200 square pixels). Fluorescent signal for hFIX was quantified as mean gray value with Fiji-ImageJ software.

### Analyses of Plasma Samples

Plasma samples from hemophilia B mice were collected by retro-orbital bleed into 0.38% sodium citrate buffer. Inhibitory antibodies to hFIX were measured by Bethesda assay as described (26). One Bethesda Unit (BU) is defined as the reciprocal of the dilution of test plasma at which 50% of FIX activity is inhibited. Measurements were carried out in a Diagnostica Stago STart Hemostasis Analyzer (Parsippany, NJ, USA). The activity of the expressed hFIX was assessed by ROX FIX chromogenic assay (Diapharma, Louisville, KY) following the manufacturer’s protocol. Enzyme-linked immunosorbent assay (ELISA)-based measurements of anti-AAV2 IgG2c, AAV1 IgG2a, and anti-FIX IgG1 antibodies were carried out as described (12, 26).

### Statistical Analysis

Results are reported as means ± standard error of the mean (SEM). Statistical significance between groups was determined by either unpaired Student’s t test or two-way ANOVA using GraphPad Prism 7 software (San Diego, CA, USA). *P* value of <0.05 was considered significant. Differences are indicated as *P < 0.05, **P < 0.01, ***P < 0.001, ****P < 0.0001.

## Results

### Capsid-Specific CD8^+^ T Cell Responses Depend on XCR1^+^ Dendritic Cells

**Figure 1:**
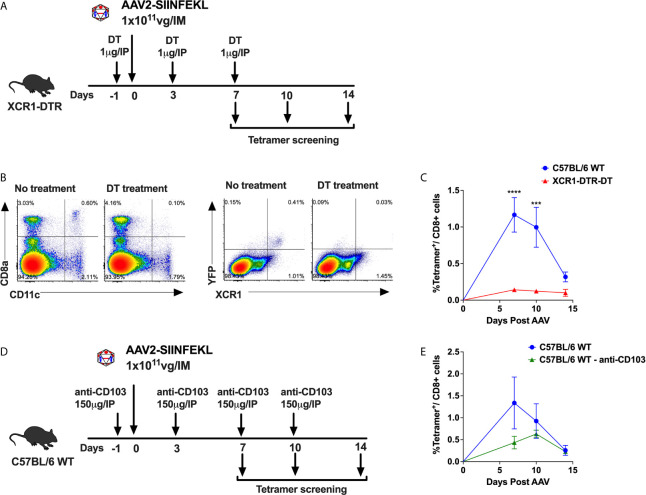
XCR1^+^ DCs required for cross-priming of capsid specific CD8^+^ T cells. **(A)** Experimental timeline showing treatment of XCR1^+/DTRvenus^ mice with DT to ablate XCR1^+^ DCs. **(B)** Representative flow cytometry plots showing CD8α^+^CD11c^hi^ DCs and XCR1^+^YFP^+^ DCs (“venus” reporter) from DT treated and untreated mice. **(C)** Anti-capsid CD8^+^ T cell response reported as percent tetramer^+^CD8^+^ T cells at 7-, 10- and 14-day time points. **(D)** Experimental timeline showing treatment of C57BL/6 WT mice with anti-CD103 antibody to neutralize CD103^+^ DCs. **(E)** Percentage of tetramer^+^CD8^+^ T cells over time in anti-CD103 antibody treated and untreated mice. Data are average ± SEM of at least five animals per group. Each circle represents an individual animal. Statistically significant differences are indicated. ***P < 0.001, ****P < 0.0001.

We have previously shown that cross-priming of AAV capsid specific CD8^+^ T cells requires cooperation of pDCs, which sense the AAV genome *via* TLR9 and produce T1 IFN, and cDCs, which sense T1 IFN and present antigen (9, 15). The cDC compartment can be divided into two main subsets, cDC1s and cDC2s which are XCR1^+^ and CD11b^+^, respectively. The cDC1 compartment can be further broken down into tissue resident CD8α^+^ DCs and migratory CD103^+^ DCs (31–35). To determine whether XCR1^+^ DCs are required for CD8^+^ T cell responses to capsid, we used XCR1^+/DTRvenus^ mice, in which XCR1^+^ resident DC can be depleted upon administration of diphtheria toxin (DT) (36). We also evaluated whether CD103^+^ DCs are necessary for anti-capsid CD8^+^ T cell responses by neutralizing CD103^+^ DCs in WT C57BL/6 mice using an anti-CD103 antibody (37). To quantify capsid specific CD8^+^ T cells, we used an AAV2 capsid containing SIINFEKL (AAV2-SIINFEKL), the immunodominant epitope of ovalbumin recognized by CD8^+^ T cells (9). AAV-capsid specific CD8^+^ T cells in peripheral blood were quantitated over time by flow cytometry using an H-2Kb SIINFEKL tetramer (9). XCR1^+/DTRvenus^ mice (on C57BL/6 genetic background) were treated with 1 µg of DT i.p. on day −1, 3, and 7. Mice not receiving DT served as positive control for the T cell response. On days 0, animals received an i.m. injection into the quadriceps with 1 × 10^11^ viral genomes (vg) of AAV2-SIINFEKL (**Figure 1A**). Upon DT injection to XCR1^+/DTRvenus^ mice, XCR1^+^ DCs (expressing the YFP “venus” reporter) and CD8α^+^CD11c^hi^ DCs (which represent the majority of XCR1^+^ DCs) were efficiently ablated as shown in **Figure 1B**. Importantly, XCR1^+^ cell depletion significantly hampered development of capsid specific CD8^+^ T cells (**Figure 1C**). In another experiment, WT C57BL/6 mice were treated with 150 µg of α-CD103 i.p. on days −1, 3, 7, and 10 to neutralize CD103^+^ DCs (**Figure 1D**). Mice treated with anti-CD103 exhibited a significantly lower frequency of tetramer^+^ CD8^+^ T cells compared with control mice (**Figure 1E**). Thus, while CD103^+^ DC neutralization produced a partial decrease of CD8^+^ T cell responses, depletion of XCR1^+^ DCs led to a complete abolishment of capsid-specific CD8^+^ T cell responses. Therefore, cross-priming of capsid-specific CD8^+^ T cells strictly relies on XCR1^+^ DCs, with the CD103^+^XCR1^+^ DC subset partially contributing.

### Depletion of Immune Stimulatory CpG Motifs Prevent AAV Capsid-Specific CD8^+^ T Cells

**Figure 2:**
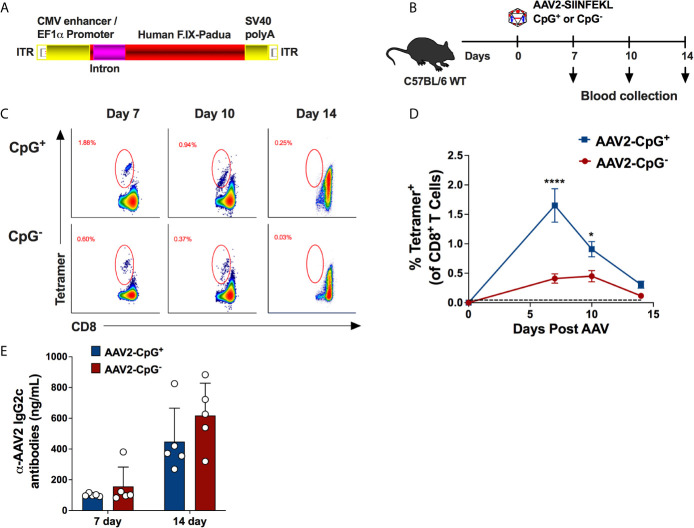
Absence of CpG motifs significantly reduce the percentage of AAV capsid specific CD8^+^ T cells but does not impact capsid-specific antibody formation. **(A)** Schematic representation of CpG-free expression cassette encoding hFIX with padua mutation. This cassette was packaged into AAV2-SIINFEKL capsid. **(B)** Experimental timeline of WT C57BL/6 mice injected with either AAV2-SIINFEKL-CpG^+^ or AAV2-SIINFEKL-CpG^−^ vector. **(C)** Representative flow plots showing AAV capsid-specific CD8^+^ T cells at different time points in mice injected with AAV2-SIINFEKL-CpG^+^ and AAV2-SIINFEKL-CpG^−^ vector. **(D)** Percentages of capsid specific CD8^+^ T cell in mice injected with AAV2-SIINFEKL-CpG^+^ and AAV2-SIINFEKL-CpG^−^ vector. The dotted line at 0.045% represents the limit of detection of capsid specific CD8^+^ T cells using the tetramer. **(E)** Anti-AAV2 IgG2c antibody titers in plasma samples from mice injected with AAV2-SIINFEKL-CpG^+^ or AAV2-SIINFEKL-CpG^−^ vector. Samples were collected at days 14 and 28 post vector injection. Data are average ± SEM of at least five animals per group. Each circle represents an individual animal. Statistically significant differences are indicated. *P < 0.05, ****P < 0.0001.

Because depleting CpG motifs from the transgene gene has been shown to mitigate AAV-immune responses (23), we hypothesized that depletion of CpG motifs from the vector genome may “deimmunize” AAV vectors and reduce the CD8^+^ T cell response. To test our hypothesis, we constructed a vector using a CpG-free expression cassette. This cassette contains a CpG-free edited sequence of the coding region for human coagulation factor IX (hFIX) and the following CpG-free elements: a CMV enhancer/EF1α promoter combination, a synthetic intron, and an SV40 polyA signal. The assembled cassette was inserted in between AAV2 ITRs and packaged into AAV2-SIINFEKL (**Figure 2A**). While this cassette contains 0 CpG motifs, the control vector with native sequences contains 41 CpG motifs (while the ITRs were unaltered in both vectors, each containing 16 CpG motifs per ITR). On day 0, WT C57BL/6 mice received 1 × 10^11^ vg of AAV2-SIINFEKL vector containing either hFIX cassette depleted of CpG motifs (AAV2-SIINFEKL-CpG^−^) or hFIX cassette containing CpG motifs (AAV2-SIINFEKL-CpG^+^; containing native sequence) *via* i.m. injection into the quadriceps muscle (**Figure 2B**). Anti-capsid CD8^+^ T cells were quantified in peripheral blood on days 7, 10 and 14. Mice injected with AAV-SIINFEKL-CpG^−^ vector had a substantially reduced frequency of tetramer^+^ CD8^+^ T cells compared to mice injected with AAV2-SIINFEKL-CpG at 7 and 10 days after vector injection (**Figures 2C, D** **)**. Low induction of anti-capsid CD8^+^ T cells by the CpG^−^ vector was also observed in a similar second experiment (**Supplementary Figure 2**). However, CpG depletion failed to prevent capsid-specific antibody formation as there was no difference in anti-AAV2 IgG2c titers between mice injected with AAV2-SIINFEKL-CpG^−^ and AAV2-SIINFEKL-CpG^+^ vector (**Figure 2E**). Thus, CpG motifs represent a critical activation signal for the generation of a CD8^+^ T cell response but not an antibody response against capsid.

### The Effect of CpG Motifs on Immune Responses to Gene Transfer in Hemophilia B Mice

**Figure 3:**
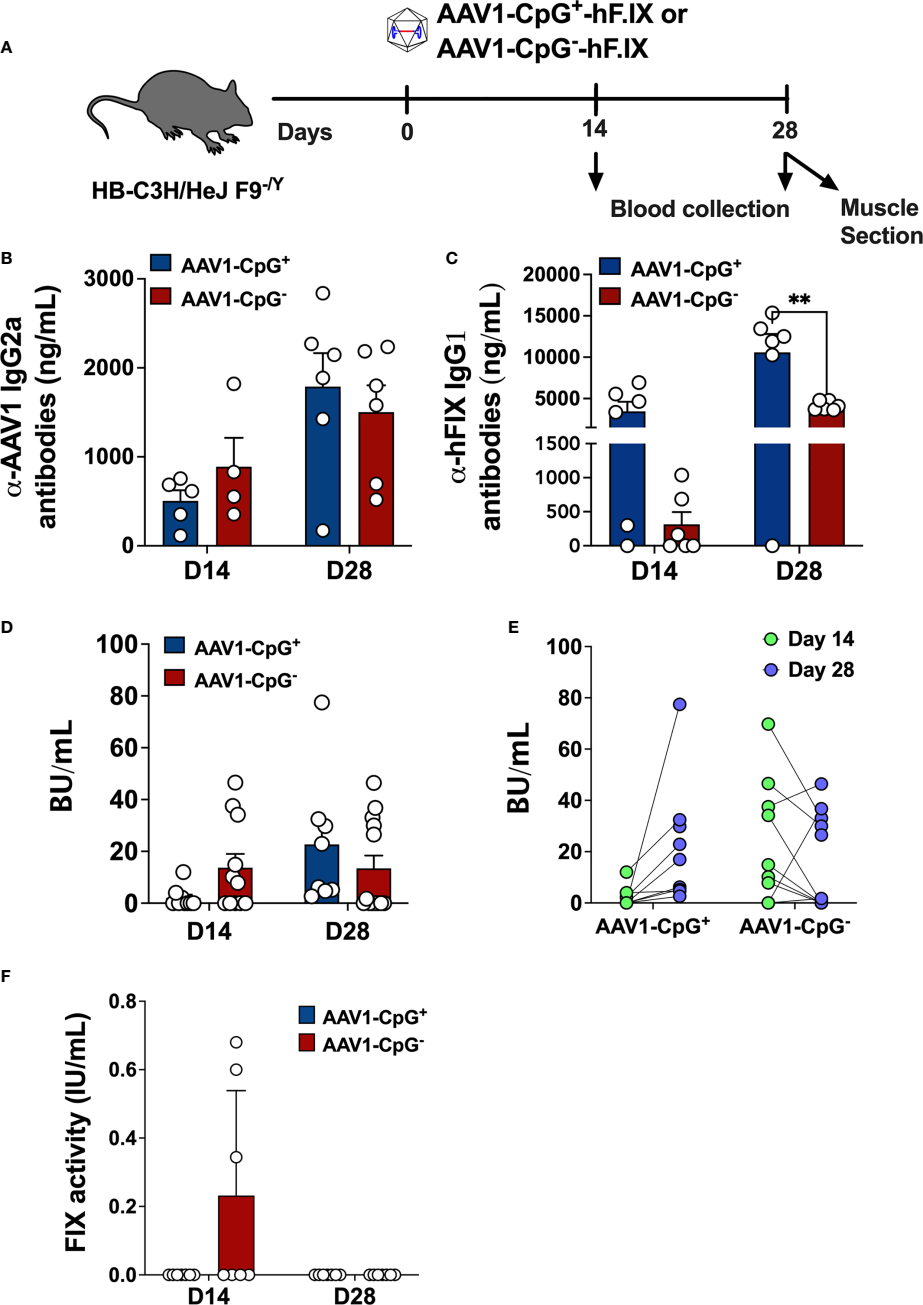
CpG depletion has no effect on antibody formation against capsid but changes the dynamics of the anti-hFIX response in hemophilia B mice. **(A)** Experimental timeline of hemophilia B (C3H/HeJ F9^−/Y^) mice injected with AAV1-CpG^+^ or AAV1-CpG^−^ vector. **(B)** Anti-AAV1 IgG2a antibody titers in plasma samples of hemophilia B mice injected with AAV1-CpG^+^ or AAV1-CpG^−^ vector. Samples were collected at day 14 and 28 post vector injection. **(C)** anti-hFIX IgG1 antibody titers anti-AAV1 IgG2a antibody titers in plasma samples of hemophilia B mice injected with AAV1-CpG^+^ or AAV1-CpG^−^ vector. **(D)** Bethesda inhibitor titers (BU/ml) in plasma samples of hemophilia B mice injected with AAV1-CpG^+^ or AAV1-CpG^−^ vector. **(E)** Correlation between inhibitor titer at different time points within the same group. **(F)** Percent hFIX coagulation activity as assessed by Rossix ROX Factor IX chromogenic assay. Data are average ± SEM of at least four animals per group and is representative of two independent experiments. Each circle represents an individual animal. Statistically significant differences are indicated. **P < 0.01.

After confirming that CpG depletion reduces AAV capsid specific CD8^+^ T cell responses, we asked whether depleting CpG motifs from hFIX expression cassette improves the outcome of gene transfer in hemophilia B mice. For that, we evaluated adaptive immune responses to hFIX in male hemophilia B (C3H/HeJ F9^−/Y^) mice injected with 1 × 10^11^ vg of AAV1-CpG^−^ or AAV1-CpG^+^ into the quadriceps (**Figure 3A**). These hemophilia B mice have a deletion of endogenous *F9* gene and therefore lack tolerance to FIX antigen (26). The immunogenic i.m. route was chosen for these experiments, as hepatic gene transfer typically results in tolerance induction to FIX (24, 38). Because the serotype AAV1, shows a superior efficiency for muscles gene transfer, AAV1 capsid was used (39). Blood samples were collected at day 14 and 28 after vector injection and antibody titers measured by ELISA. IgG2a titers (the dominant anti-capsid immunoglobulin) against AAV1 capsid were similar between groups at both time points suggesting that CpG content is less critical for anti-capsid antibody formation (**Figure 3B**). On the other hand, AAV1-CpG^−^ group showed >10-fold lower IgG1 formation against hFIX (the main IgG subclass against FIX as we had identified in our published studies) at day 14 but only ~2.5-fold lower at day 28 compared to AAV1-CpG^+^ (**Figure 3C**). Thus, CpG depletion did not impact capsid specific antibody formation, but initially reduced IgG1 formation against hFIX.

To further characterize the antibodies against hFIX, inhibitor titers were measured by Bethesda assay. At day 14, AAV1-CpG^−^ treated mice showed modestly higher incidence of inhibitor development (antibodies that inhibit FIX coagulation activity as determined by Bethesda assay); 58.3% (7 of 12) of AAV1-CpG^−^ treated mice, compared to 33.3% (3 of 9) in the AAV1-CpG^+^ injected mice (**Figure 3D**). In contrast, AAV1-CpG^+^ treated mice had increased inhibitor titers by day 28, which now were somewhat higher compared to the AAV1-CpG^−^ group (**Figure 3D**). Interestingly, when comparing the BU titers for different time points within the same group, we observed that at day 28, AAV1-CpG^−^ treated mice showed a decrease in the inhibitor formation compared to earlier time point. In contrast, AAV1-CpG^+^ mice showed an increase of inhibitor formation over time (**Figure 3E**). Although no FIX antigen was detectable in circulation (likely owing to the antibody formation), low hFIX activity of 0.3% of normal was initially detected in mice treated with AAV1-CpG^−^ (**Figure 3F**). In order to rule out that CpG depletion affected transgene expression, we also compared both vectors in a setting of immune tolerance. Hepatic gene transfer in C57BL/6 mice resulted in nearly identical levels of hFIX expression from CpG^+^ and CpG^−^ vectors, indicating that these were equally potent in hFIX transgene expression (**Supplementary Figure 3**). In conclusion, CpG motifs merely modify the time course and potency of antibody formation against the transgene product but are ultimately not required. Therefore, CpG depletion has little utility in prevention of antibody formation.

### CpG Depletion Substantially Decreased But Did Not Entirely Eliminate CD8^+^ T Cells Infiltration in Skeletal Muscle

**Figure 4:**
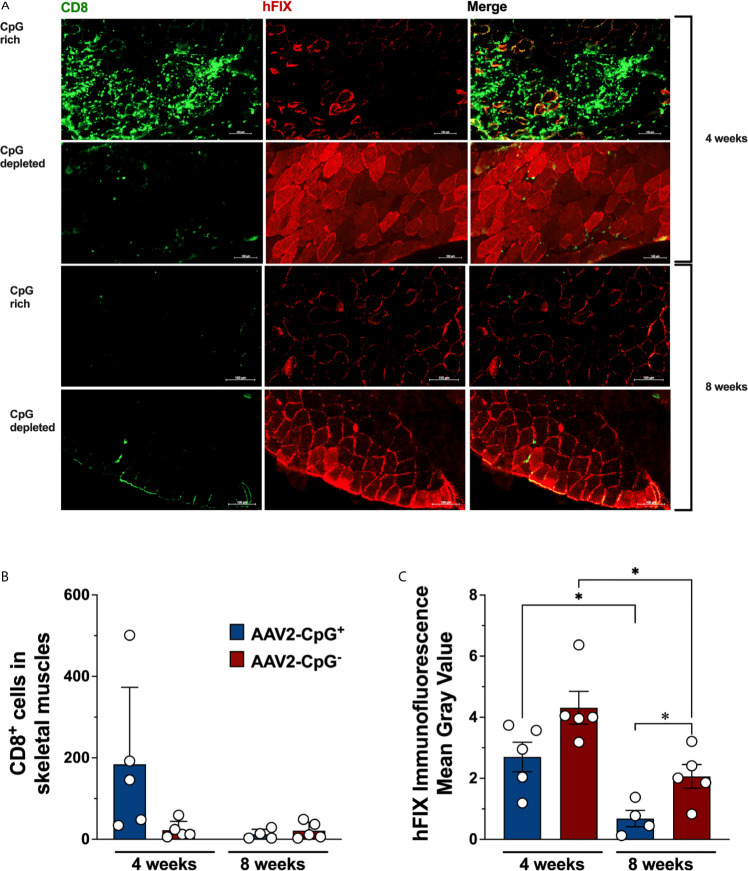
hFIX expression and CD8^+^ T cell infiltration in transduced muscles. Skeletal muscles from hemophilia B (C3H/HeJ F9^−/Y^) mice were harvested, cryosectioned, and stained for hFIX (red) and CD8 (green) 4 weeks after AAV1-CpG^−^ or AAV1-CpG^+^ injection, as described in Figure 3A. Muscles section was entirely scan and images were taken for both channels using a 40× objective with ZEISS Microscopy. **(A)** Sequential scan image of transduced muscle from AAV1-CpG^+^ (left) or AAV1-CpG^−^ (right) injected mice shown for individual channel or merge image. **(B)** Numbers of infiltrating CD8^+^ T cells in transduced muscle as quantified by Fiji-ImageJ software after generating a grid (area per point, 1200 square pixels). Whole muscle sections were quantified. **(C)** Fluorescent signal for hFIX quantified as mean gray value with Fiji-ImageJ software. Representative images from five mice are shown for each condition. The scale bar represents 100 μm. Data are average ± SEM of at least five animals per group. Each circle represents an individual animal. Statistically significant differences are indicated. *P < 0.05.

Next, we studied the effect of CpG depletion on CD8^+^ T cell infiltration in injected muscle. We know from prior experiments, that CD8^+^ T cell infiltrating AAV-hFIX transduced muscle are directed against hFIX in this strain of mouse, since hemophilic C3H/HeJ mice transgenically expressing non-functional forms of hFIX do not show this response (13, 26). We evaluated CD8^+^ T cells infiltration and hFIX expression in skeletal muscles tissue using immunofluorescence staining. Transduced quadriceps muscles of hemophilia B were harvested 28 days after gene transfer, cryosectioned, and immunostained for CD8 and hFIX. CD8^+^ T cell infiltration was consistently low in skeletal muscle of all 5 AAV1-CpG^−^ injected mice (**Figures 4A, B** **)**. In contrast, 3 of 5 AAV1-CpG^+^ injected mice showed robust CD8^+^ T cell infiltration and substantial tissue damage (**Figure 4A** and **Supplementary Figure 4**). CD8^+^ T cell infiltration in AAV1-CpG^+^ transduced muscle was on average 8-fold higher compared to AAV1-CpG^−^. This difference did not reach statistical significance because of variability in the AAV1-CpG^+^ group, where 2 of 5 animals had low levels of infiltrates. In agreement with the increased tissue damage caused by the AAV1-CpG^+^ vector, the areas of hFIX expression in cross sections of skeletal muscles was lower in AAV1-CpG^+^ injected compared to AAV1-CpG^−^ injected mice (**Figure 4C**). To confirm that CpG depletion reduced rather than merely delayed a CD8^+^ T cell response, we performed gene transfer in an additional cohort of hemophilia B mice and analyzed transduced muscles 8 weeks later. For both vectors we observed minimal CD8^+^ T cell infiltration in muscles at 8 weeks (**Figures 4A, B** and **Supplementary Figure 4**) and a reduction in hFIX expression compared to the 4-week time point (**Figure 4C**). However, AAV1-CpG^−^ vector had a significantly greater FIX expression in the muscle at 8 weeks time point compared with AAV1-CpG^−^ vector (**Figure 4C**). In summary, CpG-rich vectors were prone to causing inflammatory CD8^+^ T cell responses during the first month in transduced muscle, while CpG-depleted vectors consistently caused only mild responses. As a result, muscles transduced with CpG^−^ vector showed less tissue destruction and better preservation of transgene expression.

## Discussion

Development of immune response to the viral vector or the transgene product, particularly CD8^+^ T cell responses, represents a significant challenge for long-term success of gene therapy. DCs are responsible for the crosstalk between innate and adaptative immune systems. In case of AAV vectors, the immune response initiates with pDC sensing of hypomethylated CpG motifs in the AAV genome by TLR9. This sensing leads to T1 IFN production by pDCs. T1 IFN along with CD4^+^ T cell help contributes to licensing of cDCs, which then prime capsid specific CD8^+^ T cells (9, 15). Our new data strongly suggest that among cDCs, the XCR1^+^ subset is primarily responsible for the priming of CD8^+^ T cells. This result is consistent with the known biology of XCR1^+^ DCs, which are specialized in cross-presentation of antigen. XCR1^+^ DCs consist of CD8α^+^ and CD103^+^ DCs; and it is likely that both subsets contribute, as neutralization of the CD103^+^ subset only partially blocked the response (albeit that we cannot rule out that the antibody against CD103 was only partially effective). Others have shown that pDC-derived T1 IFN induces expression of XCR1^+^ in cDCs, optimizing their maturation, costimulatory capacity, and ability to cross-present (40). XCR1^+^ cDCs are able to present antigen on both class I and II MHC molecules, serving as a platform for simultaneous interactions between CD4^+^ and CD8^+^ T cell (41). CD4^+^ T help *via* CD40L/CD40 co-stimulation is also required for an effective CD8^+^ T cell response against AAV capsid (15). Thus, XCR1^+^ DCs, besides cross-presenting antigen to CD8^+^ T cells, establish a platform to orchestrate the cooperation between CD8^+^ T cells and CD4^+^ T help, resulting in optimal CD8^+^ T cell activation, as summarized in the proposed model in **Supplementary Figure 5**.

Pre-clinical studies have applied immunosuppression as an alternative to control anti-capsid cellular immune response (42–44). However, this may not be sufficient to prevent the loss of transgene expression, in particular if the vector is too immunogenic. *In vitro* studies showed TLR9-dependent induction of IFN I production in human pDCs pulsed with AAV vectors (8). Clinical experience with hepatic AAV gene transfer for hemophilia suggests that CpG motifs contribute to the loss of therapeutic expression and that immune suppression, when still needed, is more effective when using CpG depleted vectors (45–47). Low CpG level correlates strongly with long-term transgene expression (20). Conversely, CpG enrichment negatively affected the outcome of gene therapy for hemophilia (47, 48). Therefore, genome editing to eliminate CpG motifs and thus decrease immunogenicity of AAV vectors is a promising approach. In a proof-of-principle study, others have shown that absence of CpG sequences in the lacZ reporter transgene minimized CD8^+^ T cell infiltration and prolonged expression of a reporter transgene upon AAV gene transfer in mice (23). Curiously, Xiang et al. found that capsid-specific memory CD8^+^ T cells showed strong *in vivo* proliferative responses to AAV vectors with CpG-depleted genomes, while naive CD8^+^ T cells responded much more vigorously to CpG^+^ vectors, which provide stronger TLR9 stimulation (49). Given the above-mentioned clinical experience with these vectors, the authors concluded that responses seen in humans may mostly reflect primary responses.

Both pre-clinical studies in animal models and clinical trial data support that CD8^+^ T cell activation is linked to innate immune sensing, which serve to provide activation or “danger” signals. Here, we have refined our model for cross-priming of capsid specific CD8^+^ T cells. Sensing of CpG motifs by TLR9 in pDCs leads to T1 IFN production and activation of cross-presenting XCR1^+^ cDCs. Thus, depletion of CpG motifs lead to substantially reduced CD8^+^ T cell responses against the viral capsid and against a therapeutic transgene product. For instance, CD8^+^ T cell responses were significantly reduced in CpG^−^ transduced muscle of hemophilia B mice, thereby avoiding muscle damage and better preserving hFIX transgene expression. CpG depletion did not much affect the kinetics of the CD8^+^ T cell response but rather substantially reduced the magnitude. The reason why hFIX expression was not entirely lost in muscle of CpG^+^ treated mice may have related to the use of single-stranded AAV (ssAAV) vector. These often induce functionally impaired CD8^+^ T cells against the transgene product with a reduced cytotoxic and proliferative capacity (50), ultimately undergoing apoptosis (51). This is also the case in AAV-hFIX gene transfer to hemophilia B mice, while use of self-complementary AAV (scAAV) results in a more destructive CD8^+^ T cell response that rapidly eliminates of hFIX expressing muscles fibers (13). While we have not tested CpG depletion in scAAV vectors in this study, clinical trial results support that such a vector is also less prone to CD8^+^ T cell activation, at least against capsid (47, 52).

Our prior study in mice showed a substantial reduction in CD8^+^ T cell responses against a transgene product encoded by scAAV vector in TLR9-deficient mice (13). A quantitative method to evaluate TLR9 activation risk factors in candidate expression cassettes may be helpful to reduce AAV vector immunogenicity (22). This method has predictive potential for selected DNA sequences, thus increasing the chances for long-term clinical benefits.

However, the approach also has limitations. We still detected a residual CD8^+^ T cell response and partial loss of transduced cells. Since we only eliminated CpG motifs in the expression cassette, it is possible that CpG motifs in the inverted terminal repeats (ITRs) contributed. Also, TLR9 signaling is not entirely CpG dependent (53). For other expression cassettes, it may not be possible to remove all CpG motifs. In case of editing the promoter, one would have to empirically determine which sequences can be changed and what to change them to without altering promoter strength or specificity. An alternative approach to eliminate TLR9 signaling is to include TLR9 inhibitory DNA sequences in the vector construct, as recently been shown by Chan et al. (54). However, it should be pointed out that even TLR9 deficient mice do not show complete abolishment of CD8^+^ T cell responses, as our current and published studies have shown (7–9). Therefore, additional pathways may exist that result in CD8^+^ T cell activation. In clinical trials, CD8^+^ T cell responses have been observed against dystrophin and α1-antitrypsin in treatment of Duchenne’s muscular dystrophy and α1-antitrypsin (AAT) deficiency, respectively (55, 56). Interestingly, pre-existing cellular immunity to dystrophin may occur in some patients with Duchene’s muscular dystrophy, a disease characterized by muscle regeneration and some level inflammation (55). One should therefore caution that the underlying disease may contribute additional inflammatory signals in the target tissue of gene transfer.

Another limitation is that CpG depletion does not eliminate antibody formation against the capsid or transgene product, which can be modified by but does not depend on the TLR9-MyD88 pathway (9, 12, 15). In this new study, CpG depletion had no effect on antibody formation against capsid but changed the dynamics of the anti-hFIX response. CpG^+^ vector rapidly induced high-titer mostly non-inhibitory antibodies, which evolved into a more neutralizing response over time. CpG^−^ vector showed a lower-titer IgG1 but more focused response by day 14, with higher inhibitory titers that modestly decreased afterwards. Antibody formation against capsid was also not impacted by CpG depletion in clinical trials (19, 57).

In conclusion, our results support CpG depletion as a strategy to limit CD8^+^ T cell activation against capsid and transgene product while also pointing to limitations such as minimal impact on antibody formation and a minimized but still detectable CD8^+^ T cell response.

## Data Availability Statement

The raw data supporting the conclusions of this article will be made available by the authors, without undue reservation.

## Ethics Statement

The animal study was reviewed and approved by IACUC, Indiana University.

## Author Contributions

TB, JS, SK, IZ, and XL performed experiments. TB, JS, SK, TK, WX, and RH designed, analyzed, and interpreted experiments. TB, JS, SK, and RH wrote the manuscript. RH supervised the study. All authors contributed to the article and approved the submitted version.

## Funding

This work was supported by National Institutes of Health, National Institute of Allergy and Infectious Diseases Grant R01 AI51390, National Institutes of Health, National Heart, Lung, and Blood Institute grants R01 HL131093, R01 HL097088 to RH, U54 HL142012 to RH, and HL142019 WX, and by Indiana Collaborative Initiative for Talent Enrichment (INCITE) funds provided by Lilly Endowment to RH and WX.

## Conflict of Interest

RH served on the scientific advisory board or Ally Therapeutics and has been receiving grant funding from Luye R&D, Boston, and Spark Therapeutics.

The remaining authors declare that the research was conducted in the absence of any commercial or financial relationships that could be construed as a potential conflict of interest.
