# Supplementary material for: Effect of CpG Depletion of Vector Genome on CD8+ T Cell Responses in AAV Gene Therapy
Source: Front Immunol. 2021 May 31;12:672449. doi: 10.3389/fimmu.2021.672449 (PMC8200677; doi:10.3389/fimmu.2021.672449)
Supplement: Supplementary file 1 [file DataSheet_1.pdf]

Supplementary Figures

…atgcaaagggtcaatatgataatggctgaatcacctggcctaatcaccatctgcctactgggctacctcctgtctgctgaatgcacagt
cttcttggaccatgagaatgccaacaaaattctcaacaggcctaagagatacaactcagggaagttagaagagtttgtgcagggcaacct
agagagggagtgcatggaggaaaagtgcagctttgaggaagccagagaggtatttgagaacactgagaggaccactgagttctggaa
gcagtatgttgatggagaccagtgtgagagcaacccttgcctgaatggggggttcctgcaaagatgacatcaactcctatgagtgctggtg
cccctttggatttgagggaaagaactgtgaactagatgtcacctgcaacatcaaaaatgggaggtgtgagcagttctgcaagaacagtgc
agacaacaaggtagtctgctcttgcactgaaggttacagattagcagaaaaccagaaaagctgtgagccagcagtccccttcccctgtg
gcagggtgtctgtctcccagacctcaaagctcacaagagctgaaacagtgttccctgatgttgactatgtgaacagcacagaggctgag
acaattctggacaatatcacccagagcactcagtccttcaatgacttcaccagagtagtagggggtgaggatgccaaaccagggcagtt
cccttggcaggtggtcctcaatggaaaggtggatgctttctgtgggggctcaattgtgaatgagaagtggatagtcacagcagcccactg
tgtagaaactggtgtgaagataactgtggtagcaggggagcacaacatagaggagactgaacacacagagcagaagagaaatgtcat
caggataatccctcaccacaattacaatgcagccatcaacaaatacaaccatgatattgccctcttagagttggatgagccattggtgctca
actcatatgtgacacccatctgtatagctgacaaggaatacacaaatatcttcctgaagtttgggagtgggtatgtttcaggctgggggag
ggtgttccacaagggcaggtctgcacttgtgctgcagtacctcagggtgccacttgttgacagggccacctgcctgctgagcaccaagtt
cacaatttacaacaatatgttctgtgctgggttccatgagggaggcagagactcctgccaaggtgacagtggaggccccccatgtgactg
aggttgagggcaccagttttctaactggaataatctcttggggggaagagtgtgccatgaagggcaaatatggcatctacaccaaagtct
ccagatatgtcaactggattaaggagaagaccaagctgacctagtag…

Supplementary Figure 1: Nucleotide sequence of CpG-free version of the coding region for human FIX-Padua.

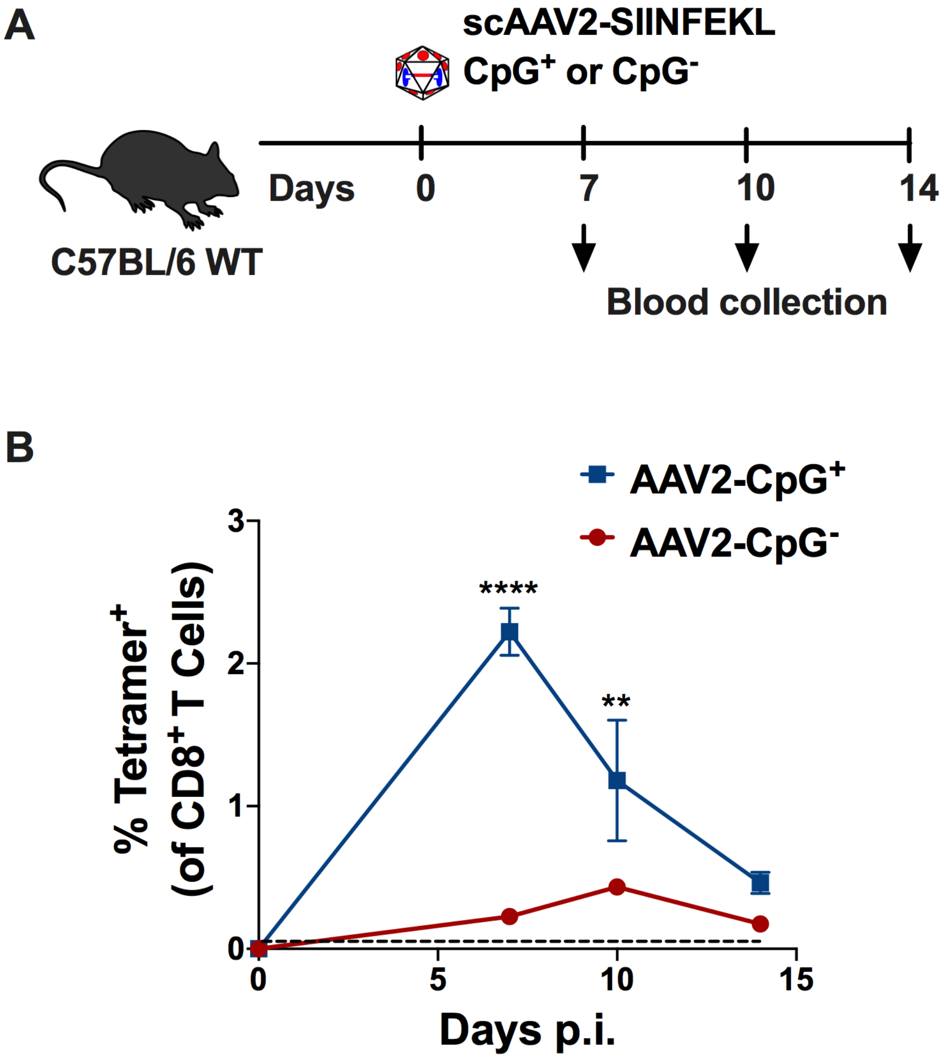

Supplementary Figure 2: Evaluation of AAV2-SIINFEKL containing CpG depleted hFIX to elicit capsid specific $CD8^+$ T cell response. A self-complementary AAV vector genome expressing GFP was used as positive control. **(A)** Experimental timeline showing AAV administration to C57BL/6 WT mice and blood collection following AAV administration. **(B)** Anti-capsid $CD8^+$ T cell response reported as percent tetramer$^+$$CD8^+$ T cells as a function of time. The dotted line at 0.045% represents the limit of detection of capsid specific $CD8^+$ T cells using the tetramer. Data are average ± SEM of at least 5 animals per group. Statistically significant differences are indicated. $^{**}P < 0.01$; $^{****}P < 0.0001$.

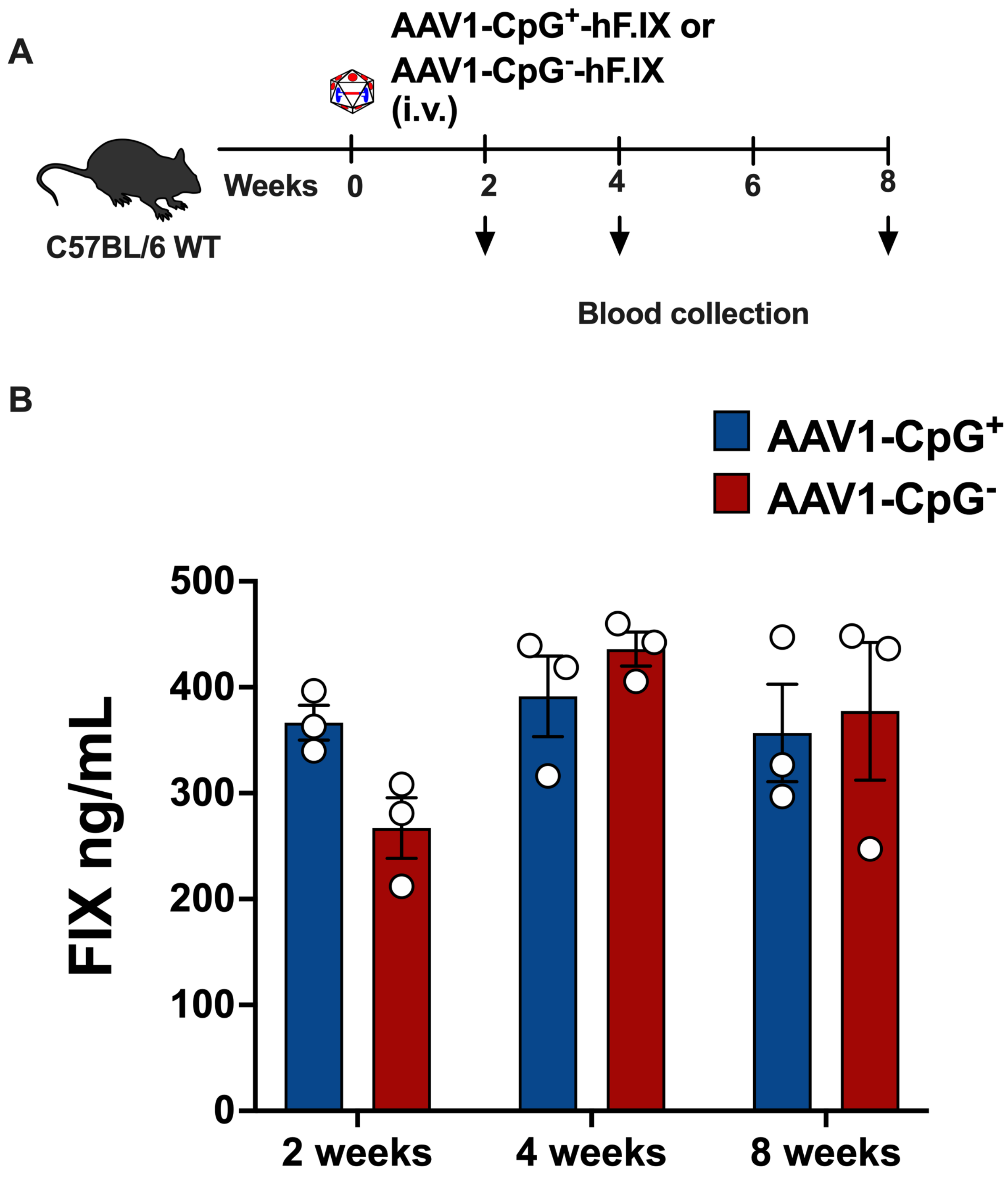

Supplementary Figure 3: CpG^+ and CpG^- vectors express similar levels of human FIX transgene product. **(A)** Experimental timeline of male C57BL/6 WT mice injected intravenously with AAV1-CpG^+ or AAV1-CpG^- vector ($1x10^{11}$ vg/animal, n=3/vector). **(B)** Human FIX levels were measured in plasma samples collected at week 2, 4 and 8 post vector injection. Each circle represents an individual animal. Data shown are average ± SEM.

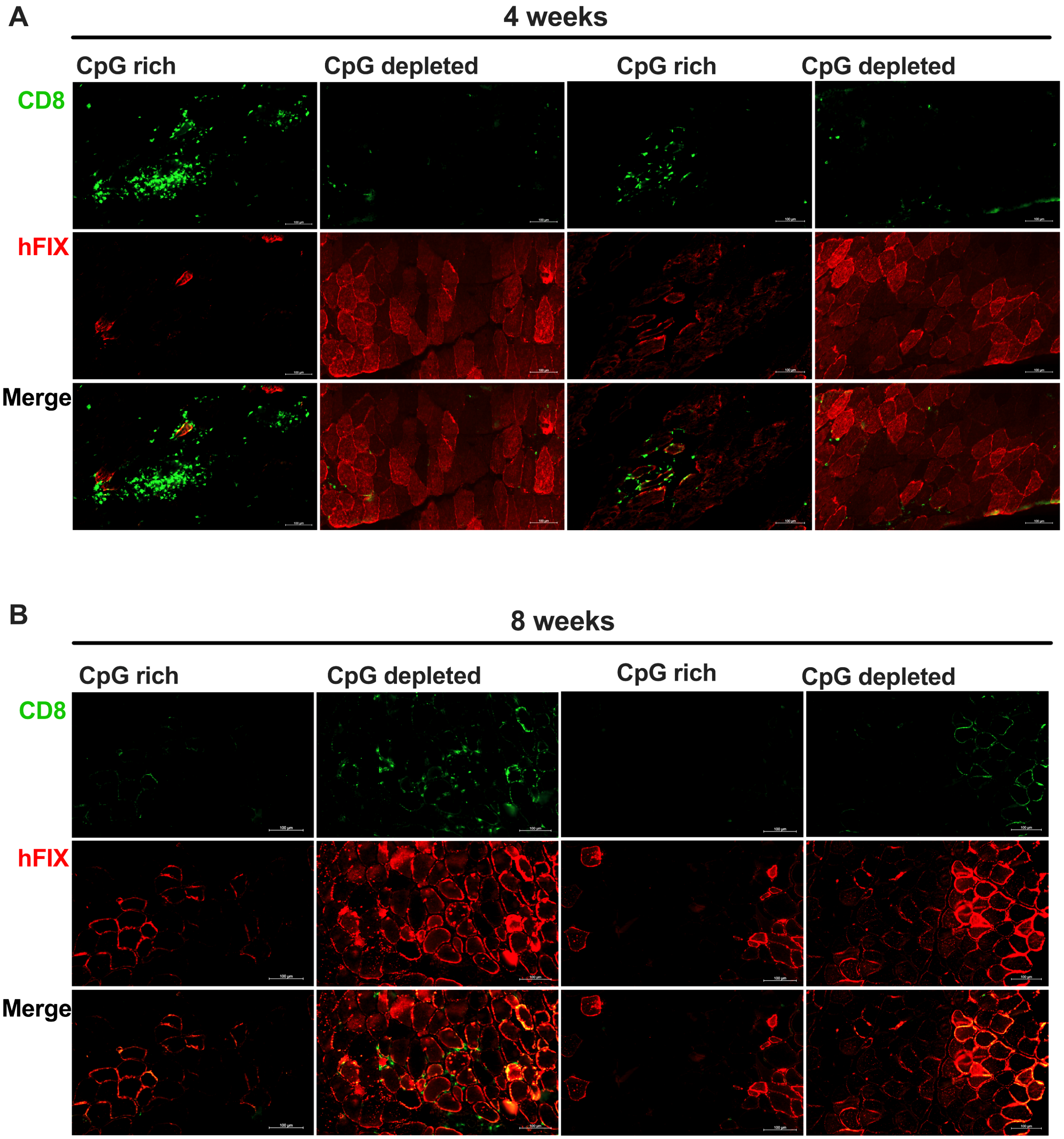

Supplementary Figure 4: Expression of hFIX (red) and infitrating CD8^{+} T cells (green) in skeletal muscle of hemophilia B mice 4 and 8 weeks after AAV1-CpG^{-} or AAV1-CpG^{+} injection. Muscles section was entirely scan and images were taken for both channels using a 40x objective with ZEISS microscope. **(A-B)** Several sequential images scan from AAV1-CpG^{+} or AAV1-CpG^{-} mice are been displayed for individual channel or merge image. Representative images are from additional individual animals of the same experiment shown in Figure 4 of the manuscript. The scale bar represents 100 µm.

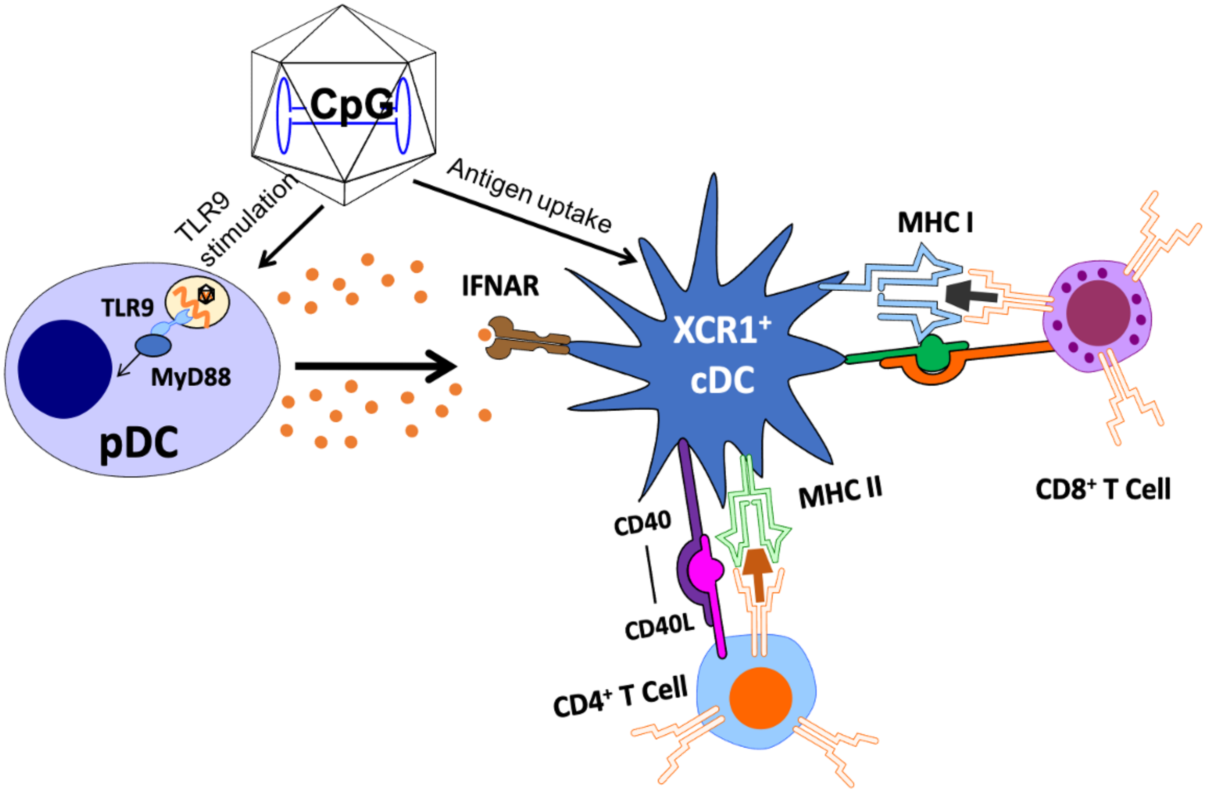

Supplementary Figure 5: Model for induction of $CD8^+$ T cell response to AAV vector or to the transgene product, which involves the cooperation of multiple immune cell types, surface receptors, and soluble mediators. DCs are responsible for sampling their environment for invading pathogens and for the crosstalk between innate and adaptative immune systems. pDCs sense unmethylated CpG motifs present in the vector's DNA genome by TLR9. Consequently, this sensing leads to T1 IFN production by pDCs. T1 IFN along with $CD4^+$ T cell help (involving CD40-CD40L co-stimulation) stimulate cDCs, leading to the activation of AAV capsid-specific $CD8^+$ T cells. The $XCR1^+$ subset is mainly responsible for the priming of $CD8^+$ T cells. $XCR1^+$ cDCs are able to present antigen on both class I and II MHC molecules, forming a platform for simultaneous interactions between $CD4^+$ and $CD8^+$ T cell.
